# Can We Further Improve the Quality of Nephro-Urological Care in Children with Myelomeningocele?

**DOI:** 10.3390/ijerph13090876

**Published:** 2016-09-01

**Authors:** Monika Miklaszewska, Przemysław Korohoda, Katarzyna Zachwieja, Michał Wolnicki, Małgorzata Mizerska-Wasiak, Dorota Drożdż, Jacek A. Pietrzyk

**Affiliations:** 1 Department of Pediatric Nephrology, Jagiellonian University Medical College, Cracow 30-663, Poland; katarzyna.zachwieja@gmail.com (K.Z.); dadrozdz@cm-uj.krakow.pl (D.D.); jacek.a.pietrzyk@gmail.com (J.A.P.); 2 Department of Electronics, Faculty of Computer Science, Electronics and Telecommunications, AGH University of Science and Technology, Cracow 30-059, Poland; korohoda@agh.edu.pl; 3 Department of Pediatric Urology, Jagiellonian University Medical College, Cracow 30-663, Poland; miwolnic@gmail.com; 4 Department of Pediatrics and Nephrology, Medical University of Warsaw, Warsaw, 02-091 Poland; mmizerskawasiak@op.pl

**Keywords:** children, myelomeningocele, chronic kidney disease, urinary tract infection, renal scintigraphy

## Abstract

Myelomeningocele (MMC) results from a failure of normal neural tube fusion in early fetal development. Retrospective, observational study of medical data of 54 children treated in Pediatric Nephrology and Urology Clinics for five years was performed. The following data were analyzed: serum creatinine, eGFR, urine analysis, renal scintigraphy (RS), renal ultrasound, and urodynamics. Mean age of studied population: 12.3 years, median of eGFR at the beginning and at the end of survey was 110.25 and 116.5 mL/min/1.73 m^2^ accordingly. Median of frequency of urinary tract infections (fUTI): 1.2 episodes/year. In 24 children: low-pressure, in 30 children: high-pressure bladder was noted. Vesicouretral reflux (VUR) was noted in 23 children (42.6%). fUTI were more common in high-grade VUR group. High-grade VURs were more common in group of patients with severe renal damage. At the end of the survey 11.1% children were qualified to higher stages of chronic kidney disease. Renal parenchyma damage progression in RS was noted in 22.2% children. Positive VUR history, febrile recurrent UTIs, bladder wall trabeculation, and older age of the patients constitute risk factors of abnormal renal scans. More than 2.0 febrile, symptomatic UTIs annually increase by 5.6-fold the risk of severe renal parenchyma damage after five years.

## 1. Introduction

Myelomeningocele (MMC) results from a failure of normal neural tube fusion in early fetal development. Including spina bifida (SB), it is one of the most common non-chromosomal birth defects, resulting in a severe damage of numerous organs. In the general population, the incidence of MMC ranges from 0.3 to 4.5/1000 births [1]. Since, in Poland, great efforts are made to treat all children born with MMC, such patients constitute a vast number of children seen by pediatric nephrologists, urologists, and primary care physicians; the affected patients require to be promptly evaluated and then subjected to long-term follow-up. In the last decade, the prognosis of MMC in children has dramatically improved, predominantly thanks to the use of superior surgical and shunt implantation techniques, combined with preventive renoprotective measures. Thus, the social and economic impact of neural tube effects is presently substantial.

The abnormal innervation of the bladder detrusor and sphincter is called a neurogenic bladder (NB). In children with MMC, it causes problems in the bladder storage and emptying functions, which may lead to high intravesical pressures and recurrent urinary tract infections (rUTIs) that, in time, will produce renal scarring and progressive deterioration of the upper urinary tract (UUT) [2]. These patients form the largest group requiring nephro-urological management, as chronic kidney disease (CKD) and renal failure (RF) affecting even 30%–40% of MMC patients are main causes of their morbidity and mortality [3].

Children with MMC are generally born with normal UUT, but are at a high risk of CKD secondary to poor bladder dynamics [4]. CKD in those patients is characterized by a highly insidious onset and course, since the diagnosis of renal impairment in MMC patients is often delayed due to a misinterpretation of serum creatinine (SCr) levels which, because of their low muscle mass, may mask kidney failure. For this reason, of vital importance is the ability to monitor their renal function using an accurate estimated glomerular filtration rate (eGFR) along with visualization and assessment of renal parenchyma in renal scintigraphy (RS) and ultrasound (US) imaging.

The aim of the study was the evaluation of the occurrence of renal parenchymal damage in RS and US examinations, as well as assessment of the renal function in children with NB secondary to MMC during a five-year survey. The secondary objective was to identify the clinical risk factors affecting those outcomes. 

## 2. Materials and Methods 

Sixty-five children treated in our center in the years 2007–2013 were initially selected to participate in the retrospective, observational study. However, 11 of them did not meet the criteria of the number and type of the required examinations. Ultimately, a detailed analysis included comprehensive medical records of 54 children followed up at the Pediatric Nephrology and Urology Clinics, Jagiellonian University Medical College, for five years. Children that, at the time of inclusion into the study, were already at the ESKD stage were excluded from the analysis. All of the included children were followed up at the above clinics for a minimum of 12 months and were treated in keeping with procedures in force and current medical knowledge. The study group included children above two years of age, since all of the available pediatric formulas for eGFR calculations were developed for older patients and, thus, there are no accurate eGFR formulas for children less than two years [5].

The study consisted of the analysis of the below listed procedures, routinely performed in our center: lab tests including determination of SCr concentration, eGFR calculation based on the one-marker equation developed by Schwartz et al. [6], general and microbiological urine analysis (performed regularly, at least once every three months), abdominal ultrasound (performed at least once every six months); renal urodynamics and ethylenedicysteine (EC) renal scintigraphy—such examinations and tests were performed once every 18–24 months; the analysis included such children in whom at least two such examinations were performed during the follow-up. In our center, caregivers of MMC children are instructed as early as possible to make note and report at the nearest visit in the clinic each case of febrile (>38.0 °C) symptomatic urinary tract infection (UTI) and the employed therapeutic method. Symptomatic UTI was defined as a positive culture of catheter-extracted urine, temperature > 38.0 °C and pyuria > 20–25 WBC/HPF [7]. 

Clinical data were entered retrospectively on a regular basis on standardized medical hospital record sheets at the time of each clinic visit. The information was then extracted between January 2007 and December 2013 for the purpose of this study.

The eGFR value was calculated based on the one-marker equation developed by Schwartz et al. [6]. eGRF calculations included only such SCr values that were determined with the child being in a good general state, without UTI episodes, or any other clinical situations that negatively affect renal function.

Renal 99mTc ethylenedicysteine scintigraphy (EC RS) is a safe, reliable, and objective method of assessing both kidney parenchymal damage, and at the same time marker stasis and secretion within the pyelocalyceal system (PCS). According to the literature, 99mTc EC RS as compared to 99mTc DMSA (dimercaptosuccinic acid) RS is an excellent single-modality comprehensive investigational agent for renal morphology, function and outflow tract evaluation with the added advantages of lower cost, convenience and low radiation exposure to the patient [8]. The analysis included examinations performed in the first or second and fourth or fifth year of the follow-up. The following parameters were evaluated: size and thickness of the parenchyma layer (1: normal; 2: decreased; 3: absence of active parenchyma layer), signs of kidney parenchymal damage (1: no parenchymal damage; 2: moderate parenchymal damage; 3: severe parenchymal damage), and marker uptake and excretion (1: proper marker uptake and excretion; 2: decelerated marker uptake and excretion; 3: absent marker uptake and excretion). Thus, a semi-quantitative analysis of the degree of kidney damage in RS was performed. The parameters were evaluated separately for each kidney and subsequently added. In further analysis, the following abbreviations were employed: RS1: no signs of kidney parenchymal damage in RS; RS2: moderate kidney parenchymal damage in RS; RS3: severe kidney parenchymal damage in RS (renal scarring). 

Urinary tract and kidney ultrasound (US) is a safe, inexpensive, widely available, and convenient method of evaluating kidney and urinary tract morphology, although it is not an objective way of assessing an organ. For this reason, in the present study, US images were interpreted by two appropriately trained and dedicated staff members. The analysis included interpretation of US examinations performed in the first and last year of the follow-up. The following parameters were evaluated: kidney parenchymal echogenicity (1: normal echogenicity; 2: increased echogenicity), corticomedullary differentiation (1: preserved; 2: obliterated), PCS stasis: stasis at the renal pelvis (1: non-dilated renal pelvises; 2: dilated renal pelvises) and the calyces level (1: non-distended calyces; 2: distended calyces). Thus, a semi-quantitative analysis of the degree of kidney damage in US was performed. The parameters were evaluated separately for each kidney and subsequently added. In further analysis, the following abbreviations were employed: US1: no signs of kidney parenchymal damage in US; US2: moderate kidney parenchymal damage in US; US3: severe kidney parenchymal damage in US.

The study included children in whom urodynamics was performed at least twice in the first or second and fourth or fifth year of the survey. While analyzing its results, attention was mainly focused on intravesical pressure, dividing the bladders into high-pressure and low-pressure (LPP ≥ 40 cm H_2_O and LPP < 40 cm H_2_O, respectively). 

All of the children included in the study were prescribed low dose chemiprophylaxis (LDCP) and clean intermittent catheterization (CIC) to be performed 2–6 times per day for a minimum of three years of the follow-up, with 21 patients employing a catheter during the night (for a minimum of two years during the follow-up). Retrograde voiding cystoureterography (RVC) was performed as clinically indicated. All the analyzed children were subjected to at least one such examination. Low-grade vesicoureteral reflux (VUR) was termed 1–3 grade VUR, while high-grade VUR was marked as 4–5 grade VUR.

## 3. Statistical Analysis

According to the most commonly used parametric description of the study group, to allow for performing a comparative study, the mean and unbiased standard deviation (SD) estimators were used to characterize experimental distribution of the data related to age, height, and weight. While height and weight proved to be Gaussian, according to the Kolmogorow-Smirnov test (threshold 0.1), the age turned out to be non-Gaussian (threshold 0.05). The test also indicated non-Gaussian distribution of percentile values for body mass and body height. Thus, for age and specified percentiles we also provided median and interquartile ranges (IQR), i.e., the range between percentiles 25% and 75%. As some of the investigated numerical data exhibited non-Gaussian distribution, for the sake of uniformity, all such sets of data were parameterized with the use of the median and IQR. The differences between distributions were tested with either the Student’s *t*-test, for data described by mean and SD, or with the Wilcoxon rank-sum test, for data summarized with the median and IQR. All of the data describing the frequency of occurrence were compared with the use of the chi-square test with Yates correction. In the comparative tests, the indication of significant difference was assumed for *p*-values below 0.05. The linear regression model comprising the relationship between the patient age and the eGFR value was built based on the McPearson correlation coefficient with a relevant *p*-value. For selected data, the receiver operating characteristic (ROC curve) analysis was performed with the smoothed curve obtained after Gaussian modelling. The STATISTICA (STATISTICA, version 10, StatSoft, Inc., Tulsa, OK, USA) and MATLAB (MATLAB, version 2015a, Mathworks, Inc., Natick, MA, USA) packages were used for all computations.

## 4. Results

The detailed characteristics of the studied population are included in Table 1.

As it follows from the data in Table 2 and Table 3 (that contain the studied parameters and risk factors of renal injury vs. individual renal damage stages assessed in RS and US respectively), the annual frequency of symptomatic urinary tract infections (fUTIs) exerted a significant negative effect on both scintigraphic and ultrasound kidney images after the five-year follow-up. Statistical significance was shown in the number of fUTI and eGFR when comparing children from the group with severe kidney damage and patients without or with moderate damage as assessed by both RS and US. There was no statistical difference between the genders in terms of studied parameters.

**Figure 1:**
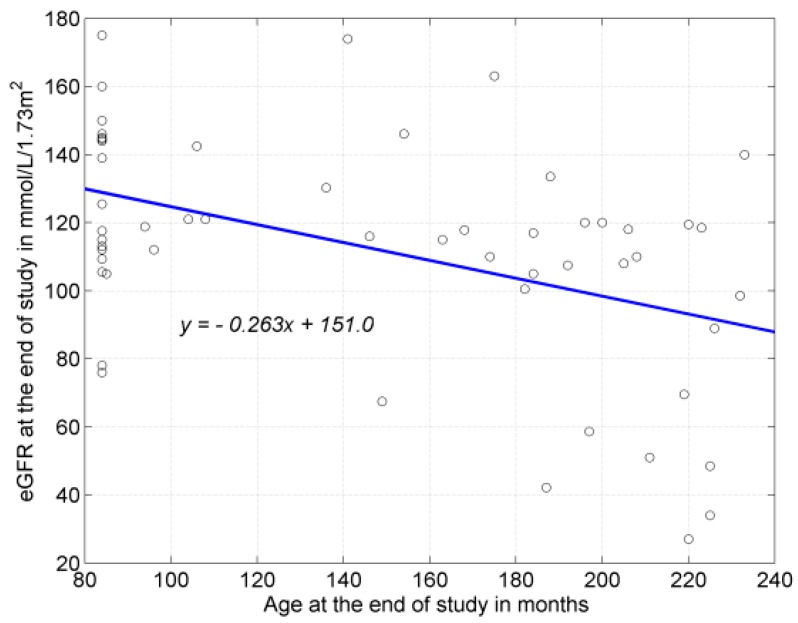
Correlation between patients age (months) vs. eGFR value at the end of five-year survey; (*r* = −0.444; *p* = 0.008).

Children with severe kidney parenchymal damage were also significantly older as compared to children without or with moderate damage (RS: 129.2 vs. 183.8 (months) (RS1 vs. 3); 140.8 vs. 183.8 (months) (RS1 + 2 vs. 3)). Moreover, a negative, average, significant correlation was noted between the age of the patients expressed in months and kidney function expressed as the eGFR value (*r* = −0.444; *p* = 0.008) (Figure 1). No correlation was observed, however, between the age of the patients and fUTI.

Additionally, high VUR values in RS assessment of kidney parenchyma function was significantly more common in the group with severe damage as compared to children without or with moderate damage (10% vs. 66.7% (RS1 vs. 3); 12% vs. 66.7% (RS2 vs. 3); 11.1% vs. 66.7% (RS1 + 2 vs. 3)). In case of US assessment, significance was noted solely between the group without signs of damage and children with severe damage. 

On the other hand, bladder wall trabeculation was significantly more common in the group with a severe or moderate degree of renal parenchymal damage as compared to patients without such damage (25% vs. 78% (RS1 vs. 3); 25% vs. 61.8% (RS1 vs. RS2 + 3)).

However, no significant differences were noted in both RS and US imaging in the percentage of high-pressure bladders, frequency of CIC employment (≥ or <4 times a day), or type of used pharmacotherapy affecting the bladder motility.

**Figure 2:**
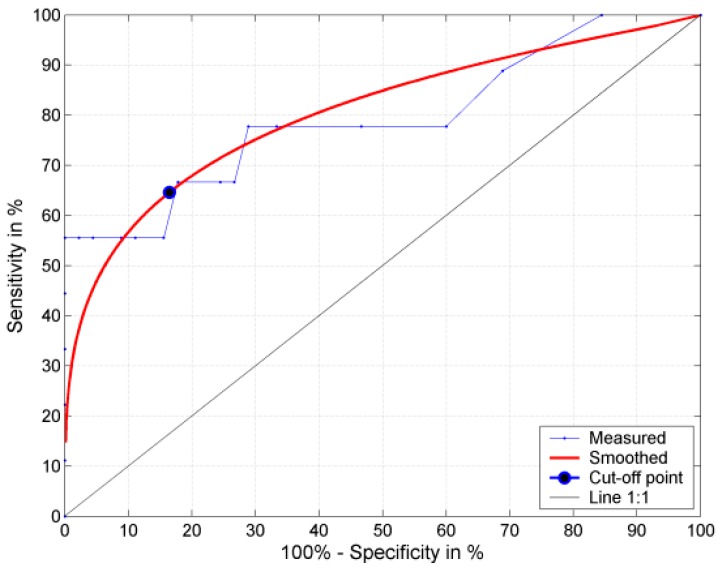
ROC curve for severe renal damage risk (RS3 vs. RS1 + 2) evaluated in RS depending on fUTI.

As it follows from the analysis of the ROC curve for fUTI after five years of the follow-up, with a cut-off value of fUTI of 2.0 annually (at 64.6% sensitivity, 83.5% specificity and 80% AUROC—what denotes a good prognostic value of the parameter), the risk of severe kidney damage in RS (RS3) increased 5.6-fold (Figure 2).

At the end of follow-up, deterioration of eGFR values was noted in 11 (30.4%) patients, of whom six (11.1%) children were qualified to higher CKD stages. Progression of renal parenchymal damage in RS imaging was seen in 12 (22.2%) children (10 showed RS1 to RS2 progression, 2—RS2 to RS3 progression); while US demonstrated such a progression in 25 (46.3%) children (14—US1 to US2 progression, 11—US2 to US3 progression). The correlation between the degree of kidney damage in RS and US imaging at the beginning and end of the follow-up was 76% and 74.1%, respectively.

As it follows from the above analysis, in the studied population, severe kidney damage was predominantly affected by an older age of the patient (both in case of imaging and renal function), higher fUTI frequency (especially fUTI > 2.0), high VUR values and the presence of bladder trabeculation indicating long-term bladder outlet obstruction. No effect on the degree of kidney damage assessed in RS or US imaging was observed in case of CIC frequency, employment of alpha-lytic and cholinergic medications, and type of neurogenic bladder.

A significantly higher fUTI value was observed in the group with high VUR grades as compared to patients without and with low VUR grades (2.2 vs. 1.0; *p* = 0.043). A significantly lower fUTI frequency was also demonstrated in the group without VUR as compared to patients with any degree of VUR (2.0 vs. 1.0; *p* = 0.033; Table 4). As it follows from the above data, the frequency of fUTI was mainly affected by high VUR grades. 

No significance was noted in fUTI in the groups with low vs. high-pressure bladders (median: 1.0 vs. 1.2) and in the groups employing CIC <4 times vs. ≥4 times/day (median: 0.8 vs. 1.2). Thus, no effect was observed of the type of neurogenic bladder and CIC frequency on the frequency of symptomatic fUTI.

Significant bacteriuria was noted in each child at least once at any time of the follow-up. However, only 33.3% cases of such significant bacteriuria were qualified as symptomatic UTI and required treatment with antibiotics or full-dose chemotherapeutic agents. Frequency of particular etiological agents of UTIs are presented in Table 5. Both the type of bacteria and their incidence in the population of MMC children as a factor in the etiology of symptomatic UTI were comparable to data from the literature [4,9].

Low dose chemiprophylaxis (LDCP) was employed in all of the children. In total, 121 treatment courses were used, including furazidin therapy in 52 (43%), trimethoprim and sulfamethoxazole in 33 (27.3%), herbal preparations in 16 (13.2%), bladder lavage (1% neomycin/gentamycin) in 10 (8.3%), fluoroquinolones (cipro-/norfloxacin) in seven (5.8%), and oral *Escherichia coli* vaccine in three (2.4%) children. One type of treatment during 5 years of the follow-up was employed in 15 (27.8%) children; two types of medications in 21 (38.9%); three types in 11 (20.4%); four types in four (7.4%); and five types in three (5.5%) patients. 

Symptomatic UTI was most commonly treated with cefuroxime (29.3%), ceftazidime (23.3%), amoxicillin with clavulanic acid (10.3%), and ciprofloxacin (9.5%). Full doses of furazidin (4.3%), meropenem (3.4%), full doses of trimethoprim and sulfamethoxazole (2.6%), piperacillin with tazobactam (2.6%), and vancomycin (2.6%) were employed more rarely. Symptomatic UTI of fungal etiology was most frequently treated with azoles (fluconazole, ketoconazole—10.3%) and caspofungin (1.8%).

## 5. Discussion

At birth, most newborns with MMC have a normal UUT. However, the majority will develop deterioration of renal function and bladder wall changes, which start within the first six months of life [2]. Two main goals in the nephro-urological management of MMC patients, which are preserving renal function and achieving social continence, are sometimes mutually exclusive since, for the UUT, sometimes it is superior to achieve a “wet but safe” rather than a “dry but unsafe” bladder. Therefore, the management of these children must be strongly focused on prevention of renal scarring and its progression to CKD.

The incidence of renal scarring in RS in the current study was 16.7%, which is quite coherent with numbers found in the literature in similar groups of patients, as several reports described scarring rates of 12.8%–28.6% [2,10,11]. Disorders of the lower urinary tract in MMC patients cause secondary VUR, and ultimately renal parenchymal damage with the contribution of UTI. Hence, several factors may contribute to the severity of renal scarring. In the current study, among others, these factors included high-grade VURs (significantly more often noted in RS3 and US3 patients), a higher frequency of symptomatic, febrile UTI (especially exceeding 2.0 episodes per year), bladder wall trabeculation, and older age of the patient. It was also noted that a higher number of fUTI was associated with high-grade VUR. In the literature, there are studies confirming that the severity of VUR and febrile recurrent UTIs affects the likelihood of renal scarring and renal deterioration [12,13]. Moreover, there are reports that the chance of cortical damage determined by RS tends to increase with age [14].

EC RS is a useful tool in determining the loss of renal parenchyma in MMC children with febrile, rUTIs and high-grade VUR. However, in case of the impossibility of performing RS, US imaging as cheap, fast, and easily accessible is also acceptable for first-line renal parenchyma assessment since, in the present investigation, the correlation between RS and US at the beginning and the end of the study was as high as 76% and 74.1%, respectively.

UTIs are a common source of morbidity among children with MMC; however, they persist as one of the most difficult complications to diagnose, treat, and prevent in those patients. Appropriate specimen collection is of a paramount importance and UTI cannot be diagnosed based on a culture and urinalysis alone in the absence of clinical symptoms. Hence, in the literature, the reported incidence of UTI in MMC patients is variable. It is estimated that the overall rate of UTI in patients with NB is 0.3 to 2.5 episodes per patient per year [15]. In the current study, the median of the number of UTI was 6.0 per patient in the 5-year follow-up, which in turn renders the frequency of UTI (fUTI) equal to 1.2 episodes per patient per year. Other studies demonstrated that the annual incidence of UTI in patients with NB ranged from 20% [16] to 36.4% [17].

Febrile, symptomatic UTIs and VURs are closely linked with each other and are strong risk factors of renal scarring and CKD progression. In the current study, VUR was noted in 42% of the patients and the percentage is quite comparable to the values reported in the literature on the subject. In the present study, the median of fUTI was statistically significantly higher in the high-grade VUR group vs. the group without VUR or with low-grade VUR, and in the patients with VUR of any grade vs. the children without this complication. The incidence of VUR in the MMC populations reported in particular studies ranges from 26.8%–50% [18]. The authors conclude that renal scars in MMC populations are associated with febrile, rUTIs, high-grade VUR, presence of bladder wall trabeculation, and older patient age. Seki et al. [19], agreeably with the current results, also noted that the presence of VUR was directly correlated with febrile UTI, while febrile UTI was a risk factor for renal scarring in MMC children. Furthermore, bladder wall trabeculation was also recognized in the literature as an independent risk factor for renal parenchymal damage and scarring [10].

According to the literature, asymptomatic bacteriuria not requiring any treatment is noted in 60%–85% of MMC patients, whereas only 21%–35% of them become symptomatic and receive pharmacological treatment [9,20]. In the current study, the percentage of symptomatic UTIs amounted to 33.3%, whereas asymptomatic bacteriuria at any time during the follow-up was noted in every patient, what may be associated with 100% of the patients being on CIC. 

The main goal of CIC is the protection of renal parenchyma by decreasing the intravesical pressure and preventing VUR. CIC, along with anticholinergics and α1-selective blockers, are commonly used treatment modalities in MMC populations worldwide. The percentage of CIC in the literature is quite variable and—to quote various authors—its number varies from 63.9%–100% of patients [3,21], whereas anticholinergics due to high bladder pressures or urinary incontinence are used according to the literature in 50%–56.1% of NB patients [15,22]. Despite their high popularity, in many studies they did not prove to be an effective treatment tool against renal deterioration in MMC patients [21,23]. Similarly, in the current study, anticholinergics, α1-selective alpha blockers, and CIC did not favorably affect the degree of renal parenchymal damage assessed in RS or US. However, considering CIC, it must be noted that this procedure during the daytime was regularly used in all our patients which, on the one hand, might have induced the strongest positive impact on renal parenchyma protection, but on the other, simultaneously happened to become a cause of a 100% incidence of asymptomatic bacteriuria in this population. Since CIC administration increases the frequency of significant bacteriuria, it simultaneously decreases the frequency of symptomatic UTI, acting as a potent renoprotective measure [24,25].

In a European survey, 85% of centers rarely prescribed LDCP to MMC children when not on CIC, but prescribed it in nearly 50% of children on CIC [22]. In the current study, all the children were on CIC and all were receiving LDCP. Studies examining the effect of LDCP on symptomatic UTI reported divergent results, with a reduction of the rate in some [26] and a higher number of infections in other cohorts [27]. Apart from that, many studies also showed that more resistant organisms may be selected out by long-term LDCP [26]. Therefore, we realize that the attitude towards long-term LDCP in our center should be reconsidered with respect to prevalence and duration of its administration. 

Despite a great progress in nephro-urological management of MMC patients, renal failure still remains life-threatening in this population. Renal function deterioration at various time frames of a long-term surveillance reported in the literature was noted, being in the ranges from 10.7%–18% [3,10] of patients, while end-stage kidney disease (ESKD) in this population was observed in the ranges from 7.5%–15% [4,28] of patients. The problem of CKD-ESKD underestimation may arise from the fact that monitoring renal function in MMC patients is particularly challenging due to a reduced muscle mass and the degree of immobility. Consequently, weight cannot be used in these patients as an indicator of lean body mass. Therefore, in MMC patients, SCr is no longer an accurate marker of renal function. Consequently, normal SCr does not exclude renal dysfunction in those patients and furthermore, renal function evaluation by SCr-based equations (like Schwartz et al. [6]) is overestimated. Similarly, in the current study, the medians of percentiles of height (12.7) and body mass (26.0) demonstrated that eGFR in this population should not be assessed using the same equations as in case of healthy children. Hence, in the present study, the outwardly median of eGFR seemed to be within normal limits and was not statistically significant when comparing the beginning and the end of the study. However, after the five-year follow-up, in particular cases, eGFR deterioration was noted in 11 (30.4%) patients, whereas six (11.1%) were qualified to higher CKD stages. Furthermore, renal parenchyma deterioration in both RS and US imaging was noted in 12 (22.2%) and 25 (46.3%) children, respectively. These results support the fact that firstly, we must neither rely on eGFR results only, nor asses the whole population in general, but also we must pay attention to renal imaging techniques while evaluating renal function in MMC patients. Moreover, a negative, statistically significant effect exerted on eGFR values was noted in case of patient age after the five-year survey.

The authors are aware of the limitations of this study. It was a retrospective, observational, single-center study, wherein all the patients were taking LDCP. Notwithstanding, during the five-year study, all the essential data were minutely collected on a current basis and the entire patient management proceeded as clinically indicated. We also realize that eGFR values calculated according to the Schwartz et al. [6] equation are overestimated. Nevertheless, in this study we concentrated on relative eGFR changes between the beginning and end of the evaluation period, hence, the bias due to eGFR overestimation did not significantly affect the final results. It seems that for a more detailed renal function evaluation in this sort of population, only a prospective study including cystatin C and iohexol assessment may yield more precise results. 

The objective of the present study was increasing our understanding of risk factors involved in renal deterioration in MMC patients. It was found that preventing febrile UTI, high-grade VUR, and bladder wall trabeculation by ensuring free urine outflow may reduce the risk of renal parenchymal damage.

To prevent renal damage, nephro-urological highly specialized management of NB should start right in the neonatal period. For optimal treatment results, a multidisciplinary approach is needed, with collaboration of members from numerous medical specialties. Compliance with long-term, wearisome treatment is suggested to be another important factor constituting a challenge for the patients and caregivers.

## 6. Conclusions 

In MMC patients, the presence of high-grade VUR is correlated with febrile UTIs. In turn, a positive VUR history, febrile rUTIs, bladder wall trabeculation and older patient age constitute risk factors of abnormal renal scans at the end of a 5 year survey in this population. More than 2.0 febrile, symptomatic UTIs annually increase by 5.6-fold the risk of severe renal parenchymal damage assessed in RS at the end of five-year follow-up. Due to the lack of reliability of SCr in MMC patients, repeated EC or DMSA RS for early detection of renal scarring are strongly recommended. However, the US imaging technique is also acceptable for general renal parenchyma assessment. 

To answer the question formulated in title of this paper, firstly: the administration of LDCP might be limited only to children with a high risk of renal cortical scarring in RS; secondly: it might be considered that renal function in this population would be assessed by the Cystatin C and iohexol method.

## Figures and Tables

**Table 1:** Clinical characteristics of the studied population (Th-L: thoracolumbar; L-S: lumbosacral; S: sacral; ACEI: angiotensin-converting-enzyme inhibitors; ARB: angiotensin receptor blockers; CCB: calcium channel blockers; BB: beta-adrenoceptor blocking agents medications; pts: patients; *: data at the end of the study).

Variable	Studied Population (*n* = 54)
Female/Male	28 (52%)/26 (48%)
Age (months); Start/End of the study; Mean (SD); (range); Median (IQR)	88.5 (57.3); (24–173)/148.0 (56.7); (84–233); 91.5 (121.0)/151.5 (116.0)
Age (years); Start/End of the study; Mean (SD); (range); Median (IQR)	7.4 (4.8); (2–14.4)/12.3 (4.7); (7–19.4); 7.6 (10.1)/12.6 (9.7)
Body mass (kg) *; Mean (SD) ; (range)	35.9 (16.4); (14–73)
Percentile of body mass *; Mean (SD); (range); Median (IQR)	26.0 (28.3); (3–90); 10.0 (47.0)
Body height (cm) *; Mean (SD); (range)	133.8 (20.3); (98–171)
Percentile of body height *; Mean (SD); (range); Median (IQR)	12.7 (18.9); (3–75); 3.0 (7.0)
Number of UTI/5 years; Median (IQR); (range)	6.0 (8.0); (2–40)
Frequency of UTI/per year; Median (IQR); (range)	1.2 (1.6); (0.4–8.0)
eGFR (mL/min/1.73 m^2^); (range); Start /End of the study; Median; (IQR); (p)	110.25 (54); (35.3–187.5)/116.5 (28.5); (27–175) (NS)
Ventriculoperitoneal shunt (%)	30 (55.6%)
Level of MMC	Th-L: 9 (16.6%); L-S: 34 (63%); S: 11 (20.4%)
Physical activity	Independent ambulation: 7 (13%)
	Needs support: 5 (9.2%)
	Wheelchair-dependent: 42 (77.8%)
Type of neurogenic bladder	Low-pressure (LPP < 40 cm H_2_O): 24
	(type I (C): 13 (24.1%),
	type II (B): 11 (20.3%)
	High-Pressure (LPP ≥ 40 cm H_2_O): 30
	(type III (A): 24 (44.4%),
	type IV (D): 6 (11.2%).
Vesicoureteral reflux (total)	23 (42.6%)
VUR: Low-grade (1–3)	12 (22.2%)
VUR: High-grade (4–5)	11 (20.4%)
CIC-Median of times a day	4-times
6 times a day	12 patients
5 times a day	12 patients
4 times a day	15 patients
3 times a day	7 patients
2 times a day	8 patients
Overnight catheter	21 patients
Alpha-1 blockers (minimum for 18 months)	18 (33.3%)
Cholinolytics (minimum for 18 months)	36 (66.7%)
Number of children with arterial hypertension	13 (24.1%)
Arterial hypertension treatment	ACEI:13; ARB:3; CCB-2; BB:2, Diuretics:2

**Table 2:** Univariate analysis of RS findings in relation to various risk factors.

Studied Variable	RS1 (*n* = 20) (37%)	RS2 (*n* = 25) (46.3%)	RS3 (*n* = 9) (16.7%)	RS1 + 2 (*n* = 45) (83.3%)	RS2 + 3 (*n* = 34) (63%)	P: RS1 vs. RS2	P: RS1 vs. RS3	P: RS2 vs. RS3	P: RS1 + 2 vs. RS3	P: RS2 + 3 vs. RS1
Age (months) Mean (SD)	129.2 (53.3)	150.1 (58.3)	183.8 (45.1)	140.8 (56.5)	159.0 (56.5)	NS	0.013	NS	0.037	NS
eGFR (mL/min/1.73 m^2^) Median (IQR)	117.4 (27.8)	118.5 (31.0)	67.5 (43.1)	118.0 (29.4)	114.0 (45.0)	NS	0.001	0.003	0.001	NS
fUTI Median (IQR)	0.90 (1.00)	1.20 (1.10)	4.00 (5.30)	1.00 (1.25)	1.30 (1.40)	NS	0.010	0.012	0.006	NS
VUR (high-grade) (%)	2 (10%)	3 (12%)	6 (66.7%)	5 (11.1%)	9 (26.5%)	NS	0.007	0.006	0.001	NS
Bladder Trabeculation (%)	5 (25%)	14 (56%)	7 (78%)	19 (42.2%)	21 (61.8%)	NS	0.024	NS	NS	0.020
LPP ≥ 40 cm H_2_O	8 (40%)	15 (60%)	7 (77.8%)	23 (51.1%)	22 (64.7%)	NS	NS	NS	NS	NS
CIC ≥ 4 times a day	14 (70%)	18 (72%)	7 (77.8%)	32 (71.1%)	25 (73.5%)	NS	NS	NS	NS	NS
α1 blockers	7 (35%)	9 (36%)	2 (22.2%)	16 (35.6%)	11 (32.4%)	NS	NS	NS	NS	NS
Cholinolytics	14 (70%)	17 (68%)	5 (55.6%)	31 (68.9%)	22 (64.7%)	NS	NS	NS	NS	NS
Both α1 blockers and Cholinolytics	6 (30%)	6 (24%)	2 (22.2%)	12 (26.7%)	8 (23.5%)	NS	NS	NS	NS	NS
Neither α1 blockers nor Cholinolytics	5 (25%)	5 (20%)	4 (44.4%)	10 (22.2%)	9 (26.5%)	NS	NS	NS	NS	NS

**Table 3:** Univariate analysis of US findings in relation to various risk factors.

Studied Variable	US1 (*n* = 21)	US2 (*n* = 18)	US3 (*n* = 15)	US1 + 2 (*n* = 39)	US2 + 3 (*n* = 33)	P: US1 vs. US2	P: US1 vs. US3	P: US2 vs. US3	P: US1 + 2 vs. US3	P: US2 + 3 vs. US1
Age (months) Mean (SD)	128.7 (51.8)	141.4 (58.8)	182.9 (47.0)	134.5 (54.8)	160.2 (57.0)	NS	0.003	0.035	0.004	0.045
eGFR (mL/min/1.73 m^2^) Median (IQR)	119.5 (28.4)	118.2 (30.7)	69.5 (67.6)	118.8 (29.8)	112.0 (45.9)	NS	0.004	0.006	0.001	NS
fUTI Median (IQR)	1.00 (1.45)	1.10 (0.80)	2.00 (3.80)	1.00 (1.10)	1.20 (1.65)	NS	0.025	0.015	0.008	NS
VUR (high-grade) (%)	1 (4.8%)	4 (22.2%)	6 (40%)	5 (12.8%)	10 (30.3%)	NS	0.027	NS	NS	NS
Bladder Trabeculation (%)	6 (28.6%)	8 (44.4%)	12 (80%)	14 (35.9%)	20 (60.6%)	NS	0.007	NS	0.009	0.044
LPP ≥ 40 cm H_2_O	10 (47.6%)	9 (50%)	11 (73.3%)	19 (48.7%)	20 (60.6%)	NS	NS	NS	NS	NS
CIC ≥ 4 times a day	13 (61.9%)	13 (72.2%)	13 (86.7%)	26 (66.7%)	26 (78.8%)	NS	NS	NS	NS	NS
α1 blockers	9 (42.9%)	7 (38.9%)	2 (13.3%)	16 (41%)	9 (27.3%)	NS	NS	NS	NS	NS
Cholinolytics	13 (61.9%)	13 (72.2%)	10 (66.7%)	26 (66.7%)	23 (69.7%)	NS	NS	NS	NS	NS
Both α1 blockers and Cholinolytics	7 (33.3%)	5 (27.8%)	2 (13.3%)	12 (30.8%)	7 (21.2%)	NS	NS	NS	NS	NS
Neither α1 blockers nor Cholinolytics	6 (28.6%)	3 (16.7%)	5 (33.3%)	9 (23.1%)	8 (24.2%)	NS	NS	NS	NS	NS

**Table 4:** Univariate analysis of fUTI in relation to various grades of VUR.

fUTI	VUR: No (*n* = 31)	VUR: High-Grade (*n* = 11)	VUR: No + Low-Grade (*n* = 43)	VUR: Low + High-Grade (*n* = 23)	P1: VUR: No vs. Low + High-Grade	P2:VUR No + Low-Grade vs. High-Grade
fUTI Median (IQR)	1.0 (0.9)	2.2 (3.9)	1.0 (1.4)	2.0 (2.1)	0.033	0.043

**Table 5:** Frequency of particular etiological agents of UTI in the studied population.

Ethiological Agent	Percentage of UTI (%)
Enterobacteriaceae family (Gram negative)	
*Escherichia coli*	36.9
*Klebsiella* spp. (pneumoniae, oxytoca)	16.8
*Proteus* spp. (vulgaris, mirabilis)	4.0
*Enterobacter* spp. (cloacae, agglomerans, hafniae)	3.0
*Citrobacter* (koseri, freundii)	1.7
*Morganella morganii*	1.3
*Serratia marcescens*	0.7
Non-Enterobacteriaceae family (Gram negative)	
*Pseudomonas* spp. (aeruginosa, putida)	21.6
*Acinetobacter baumanii*	1.3
*Stenotrophomonas maltophilia*	0.7
Cocci (Gram positive)	
*Enterococcus* spp. (faecium, faecalis)	5.1
*Staphylococcus aureus*	1.3
Yeasts	
*Candida* spp. (albicans, parapsilosis, tropicalis, krusei)	5.6

## Abbreviations

The following abbreviations are used in this manuscript:

MMC	Myelomeningocele
NB	Neurogenic Bladder
SB	Spina Bifida
rUTI	recurrent Urinary Tract Infections
VUR	Vesicoureteral Reflux
ESKD	End Stage Kidney Disease
CKD	Chronic Kidney Disease
LDCP	Low Dose Chemoprophylaxis
LPP	Leak Point Pressure
UUT	Upper Urinary Tract
